# Effect of Huoxiang Zhengqi Pill on Early Neurological Deterioration in Patients with Acute Ischemic Stroke Undergoing Recanalization Therapy and Predictive Effect of Essen Score

**DOI:** 10.1155/2020/6912015

**Published:** 2020-09-09

**Authors:** Zhi-Xin Huang, Jianguo Lin, Cheng Zhang, Ying-Yi Dai, Songbin Lin, Xintong Liu

**Affiliations:** ^1^Department of Neurology, Guangdong Second Provincial General Hospital, Southern Medical University, Guangzhou 510317, China; ^2^Department of Neurology, The Second School of Clinical Medicine, Southern Medical University, Guangzhou 510317, China; ^3^P3 Biosafety Laboratory, Guangdong Second Provincial General Hospital, Southern Medical University, Guangzhou 510317, China

## Abstract

Early neurologic deterioration (END) in the acute phase of ischemic stroke is a serious clinical event, which is closely related to poor prognosis. Therefore, it is important to identify presentation features that predict END and take relevant treatment measures, as they could help to prevent the deterioration of high-risk patients. The prospective intervention study was carried out from January 2018 to December 2019. We included consecutive patients hospitalized for acute ischemic stroke (AIS) within 6 hours of onset. Patients were randomly assigned (1 : 1) to recanalization therapy plus Huoxiang Zhengqi Pill (HXZQ) (intervention group) or standard recanalization therapy alone (control group). The primary outcome was the development of END according to predefined criteria within the first 1 week of stroke onset. Poisson regression was used to identify predictors for END. Of the 155 patients enrolled in the study (age, 63 ± 11 years; 28.4% female), 20 (12.9%) developed END. Univariate analysis showed that the use of HXZQ and Essen stroke risk score (ESRS) (low risk group) were protective factors for END, while advanced age was a risk factor for END. However, in multivariate analysis, only ESRS (OR, 0.232; 95%CI, 0.058–0.928; *P*=0.039) and the use of HXZQ (OR, 0.297; 95%CI, 0.096–0.917; *P*=0.035) were statistically significant. ESRS can be used as the prediction factor of END. HXZQ has small side effects and wide indication. It could be used in the treatment of AIS.

## 1. Introduction

Stroke is a leading cause of long-term disability and death in China, of which about 70% are ischemic stroke [1]. Intravenous thrombolysis with recombinant tissue plasminogen activator within 4.5 hours after onset has been proved to be effective against acute ischemic stroke (AIS) and can reduce mortality. However, only a small number of patients were treated within the time window, and some patients still experienced neurologic deterioration after thrombolysis, which is called early neurologic deterioration (END) [2]. END is one of the most common complications of AIS, affecting about a third of ischemic stroke patients and increasing the risk of adverse functional outcomes and death [3–5]. Therefore, it is necessary to investigate the likely impact on the prediction and treatment strategies for END and to provide a novel strategy for the diagnosis and treatment of AIS.

Previous studies have found that coronary artery disease is usually associated with severe intracranial and extracranial atherosclerosis and that progressive neurological deterioration is more common in patients with coronary artery disease or carotid artery stenosis. This finding suggests that patients with extensive atherosclerosis are more likely to develop cerebrovascular disease and are more likely to have progressive stroke [6, 7]. It is therefore necessary to explore whether the Essen stroke risk score (ESRS) (including atherosclerotic risk factors, such as hypertension, diabetes, and smoking) can be used to predict END.

Huoxiang Zhengqi Pill (HXZQ) is mainly composed of *pinellia ternata*, tangerine peel, licorice, and so on, which has been applied to treat all kinds of diarrhea in China for thousands of years. Pharmacological studies have shown that composition is roughly the same as Erchen decoction, and their main components (such as licorice) have antiplatelet aggregation [8], antioxidative [9], and neuroprotective effects [10]. The main bioactive substances of licorice are glycyrrhizin and its aglycone glycyrrhetinic acid, which implicated in antioxidation, anti-inflammation, and neuroprotection [11, 12]. The regulatory effect of glycyrrhizin on gap junction channels (GJCs) has been well demonstrated in the previous studies. Because the electrical activity in the brain comes from neuronal communication, GJCs not only act as ion-transmitting neurons, but also distribute energy flow between cells in different cell types. Stressed central nervous system cells restore their derailment by increasing the local expression of gap junction proteins in damaged neurons and glial cells [13, 14]. Therefore, the aim of this study was to explore the predictors of END and to observe the preventive and therapeutic effects of HXZQ on END.

## 2. Methods

### 2.1. Research Design and Inclusion Criteria

This study is a single-center, prospective, open-label, randomized controlled research to blindly evaluate end-point events. The study was supported by the ethics committee of the hospital (No. GD2H-QR-KJ-018). This study was conducted to evaluate the effect of HXZQ on AIS diagnosed by computed tomography (CT) within 6 hours after onset. Subjects were randomly assigned to HXZQ (Beijing Tongrentang, Z13022498) treatment and control groups. Patients with arterial occlusion confirmed by CT angiography, magnetic resonance angiography, or digital subtraction angiography should be admitted for endovascular treatment. Criteria for selecting the subjects were as follows [15]: (1) prestroke mRS Score 0-1, (2) age ≥ 18 years old, (3) treatment could be started within 6 hours of onset (inguinal puncture), and (4) informed consent of patients and/or agents. Exclusion criteria: (1) intracranial hemorrhage, (2) significant mass effect (midline shift) or infarct size was larger than 1/3 of middle cerebral artery, (3) infarct volume more than 50–100 ml (4) ASPECTS less than 8 point, (5) heart, liver, kidney, and other organ failure, and (6) pregnancy and malignant tumor.

From January 2018 to December 2019, we successively enrolled 160 patients with AIS in our center, of which 2 cases refused to participate in the study, 3 cases had incomplete imaging data, and a total of 155 cases were included. One neurologist and one radiologist evaluated the lesion vessels, which were divided into anterior circulation and posterior circulation, and were included in the posterior circulation analysis if the main lesion vessels were both anterior and posterior circulation.

Demographic data were recorded on admission, including age, sex, and history of hypertension (defined as self-reported hypertension, oral antihypertensive drugs on admission, systolic blood pressure ≥ 140 mmHg, and/or diastolic blood pressure ≥ 90 mmHg), diabetes (including self-reported diabetes or oral hypoglycemic drugs, or insulin injection), coronary heart disease, and smoking status. In addition to biochemical indicators, the National Institutes of Health Stroke Score (NIHSS), blood pressure (average value of 3 consecutive measurements), and ESRS were recorded for each patient on admission.

The primary outcome was END, defined as an increase in NIHSS score (1–3 times per day) of ≥2 points within 7 days after admission [16].

### 2.2. Statistical Analysis

According to our previous research, the incidence of END in our center is about 30%. Compared with the control group, the incidence rate of END decreased by 40% in the HXZQ treatment group with 80% power (*α* = 0.05). PASS 11.0 software (NCSS LLC: Kaysville, UT, USA) was used to calculate the sample size of 150 patients with 75 cases in each group.

Numerical data were expressed as the mean ± standard deviation or the median (quartile spacing), and categorical variables were expressed by frequency (percentage). The Shapiro-Wilk test was used in the normal test. Univariate Poisson regression analysis was performed to compare the baseline characteristics of the two groups according to whether the END occurred or not. Fisher's exact test was used if the theoretical frequency ≤1. Finally, the independent variables with *P* value less than 0.15 in univariate analysis were included in multivariate analysis. Statistical analyses were performed with the SPSS version 25.0 software package (SPSS IBM Inc, Chicago, IL, USA). *P* value <0.05 was regarded as statistically significant.

## 3. Results

A total of 155 patients with AIS within 6 hours of onset were enrolled. The average age was 63 years, with 44 females (28.4%). The main lesions of anterior and posterior circulation were 111 cases (71.6%) and 44 cases (28.4%), respectively. The low-risk group (0–2 ESRS) and the high-risk group (≥3 ESRS) were 94 (60.6%) and 61 (39.4%) cases, respectively, and 20 cases (12.9%) were diagnosed as END within 7 days after admission. Most patients were treated with antiplatelet (89.7%) and statins (95.5%), and 77 cases (49.7%) were treated with HXZQ. The baseline characteristics of the patients are shown in Table 1.

**Figure 1:**
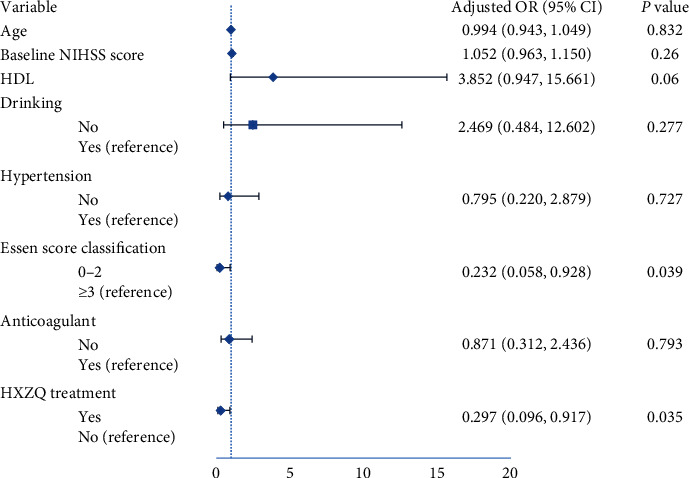
The multivariable Poisson model identifying predictors of early neurological deterioration.

Univariate analysis showed a significant difference in age (odds ratio (OR) 045; 95% confidence interval (CI) 1.006–1.085; *P*=0.024), HXZQ use (OR 0.253; 95% CI 0.085–0.757; *P*=0.014), and ESRS (low-risk group vs medium-high-risk group: OR 0.162; 95% CI 0.054–0.485; *P*=0.001) between the END group and non-END Group, in which the END group was older than the non-END Group, while the incidence of END was lower in HXZQ and ESRS low-risk groups (Table 1). As shown in Figure 1, in multivariate Poisson analysis, controlling for confounding factors, the incidence of END was lower in the low risk (ESRS) group and decreased in the HXZQ group; however, there was no statistical significance in age.

## 4. Discussion

END is a common and early event and is associated with poor prognosis in early-stage AIS. Therefore, the study of END predictors and the selection of appropriate therapy are related to the prognosis of AIS. Our findings indicated that patients with low-risk Essen scores have a low incidence of END, and oral administration of HXZQ may reduce the incidence of END.

In this study, END was present in 12.9% of AIS patients, which were similar to previously published studies (9.9% [17] and 13.5% [18]). One important clinically relevant finding was patients with a high risk in the ESRS are first reported to be more susceptible to END, a possible mechanism that links ESRS to END as a risk factor for cerebrovascular disease: first, previous studies have found that long-term hyperglycemia leads to lactic acid accumulation, promotes mitochondrial dysfunction, and aggravates the injury of AIS neurons, cerebral hypoperfusion, cerebral edema, and hemorrhagic transformation [19, 20]. Other studies have shown that diabetes causes an increase in metalloproteinase-9, which leads to increased permeability of the blood-brain barrier and infiltration of inflammatory cells, resulting in a poor prognosis of AIS. In addition, the increased hypercoagulability is also the key to vascular complications in patients with type 2 diabetes and is associated with poor prognosis in AIS patients. These are the potential mechanisms for poor prognosis after AIS in diabetic patients [21, 22]. Secondly, hypertension can lead to the abnormal dilation of cerebral microvessels in the ischemic area, which weakens the self-regulation function of cerebral vessels in this area and then leads to the increase of blood flow and damage of blood-brain barrier. In addition, when blood pressure decreased after successful recanalization, microvascular self-regulatory dysfunction may exacerbate the hypoperfusion of the penumbra [23]. Blood pressure may be involved in the development of the END by changing the state of the hemodynamics, and the decrease of cerebral hemodynamic reserve and poor collateral circulation may be related to END. Therefore, early treatment of stenosis and control of arteriosclerosis can improve cerebral perfusion and thus prevent the progression and recurrence of cerebral infarction.

To the best of our knowledge, another important finding was the first report that HXZQ can reduce the incidence of END in patients with AIS. The theory of traditional Chinese medicine believes that excessive fat is easy to produce dampness and phlegm, while turbid phlegm generates the disorder of a spleen and stomach's transportation. As a result, the body cannot absorb the essence of food or transport body fluid. If the body fluid accumulates, it is easy to produce phlegm, which can lead to vascular blockage. Animal studies showed that Erchen decoction (the same ingredients as HXZQ) could regulate the caveolae mRNA expression in rats fed with high-fat diet, lower the blood glucose, regulate the lipid metabolism, and reduce the insulin resistance; it also increased the expression of CDKAL1 MRNA and protein in mouse liver and subcutaneous fat and promoted the secretion of insulin [24]. In addition, glial cells first respond to AIS by upregulating transcription of early proinflammatory cytokines, such as TNF-*α*, leading to neuronal death and releasing neurotoxic substances into the bloodstream, further enhancing metalloproteinase expression [25, 26]. Moreover, animal experiments confirmed that TNF-*α* is closely related to acute brain edema [27]. In short, the upregulation of metalloproteinases may be a part of the vascular wall response induced by inflammatory stimuli (TNF-*α*) triggered by AIS. HXZQ can inhibit the expression of TNF*α*; therefore, it has a preventive and therapeutic effect on END [28].

Tangerine peel, one of the main components of HXZQ, contains more polymethoxy flavones, which plays a neuroprotective role by interacting with signal transduction pathways and/or altering cerebral vascular blood flow, including (1) alleviation of neurotoxin-induced neuronal damage, (2) inhibition of neuroinflammation, and (3) protecting of learning, memory, and cognition [29]. Thus, it is possible to prevent and treat END. In addition, licorice has biochemically active substances including glycyrrhizic acid and liquiritin. Glycyrrhizic acid protects neural tissue from hypoxic injury in vitro by modulating the PI3K/Akt pathway and inhibiting the glutamate-NMDA mediated neurotoxicity [30, 31]. It is reported that glycyrrhizin also plays a key role in axonal regeneration and nerve tissue repair [32]. Moreover, licochalcone was also reported to reduce platelet activation in experimental animals and humans by inhibiting cyclooxygenase-1 (COX-1) activity [33] and collagen-induced platelet aggregation [34]. These results indicated that benefits of HXZQ could be considered as a potential therapeutic agent to control or limit END.

There are some limitations in the present study. First, the study is a single-center inpatient study with a small sample size, and the results need to be further validated in a large multicenter prospective study. Second, although the attending neurologists believe that endovascular therapy should be initiated immediately if the lesion vessels fail to recanalize after thrombolysis, the manner of reperfusion therapy is inconsistent. Third, the criteria for defining END have been inconsistent in the degree or time frame of neurological deficits and are still evolving [35]. In the present study, END was defined as an increase of 2 points or more in NIHSS score within 72 hours after admission. This criterion is used because it minimizes interevaluator variations and distinguishes END from late deterioration mainly caused by stroke complications such as recurrent stroke or aspiration pneumonia [36].

## 5. Conclusions

In conclusion, ESRS could be used as a predictor factor of END. While HXZQ, as a kind of Chinese herbal medicine, has the characteristics of multicomponent and multitherapeutic target, which can reduce the occurrence of END and is suitable for the treatment of AIS in acute stage. A further study could assess the long-term effects of HXZQ in a large-scale prospective randomized controlled trial.

## Figures and Tables

**Table 1:** Comparison of variables between all patients with and without END.

Variable	All (*n* = 155)	Unadjusted OR	95% CI	*P* value
Demographics				
Age, mean (SD), years	63 (11)	1.045	1.006–1.085	0.024
Female, *n* (%)	44 (28.4)	0.925	0.355–2.407	0.873
BMI, Median (IQR)	23.7 (21.5–25.4)	1.043	0.902–1.206	0.568

Clinical characteristics				
Systolic blood pressure Median (IQR), mm Hg	142 (130–158)	0.991	0.971–1.012	0.409
Diastolic blood pressure, Median (IQR), mm Hg	85 (76–92)	0.990	0.955–1.027	0.584
Sleeping well, *n* (%)	143 (92.3)	0.755	0.175–3.255	0.706
Dysphagia, *n* (%)	43 (27.7)	1.536	0.513–4.594	0.443
Baseline NIHSS score, median (IQR)	6 (2–11)	1.063	0.985–1.147	0.116

Essen score classification, *n* (%)				
0–2	94 (60.6)	0.162	0.054–0.485	0.001
≥3	61 (39.4)	Reference		

Cerebral infarction site, *n* (%)				
Anterior circulation	111 (71.6)	0.925	0.355–2.407	0.873
Posterior circulation	44 (28.4)	Reference		

Medical history				
Smoking, *n* (%)	70 (45.2)	0.654	0.261–1.639	0.365
Drinking, *n* (%)	41 (26.5)	0.309	0.072–1.332	0.115
Hypertension, *n* (%)	98 (63.2)	2.327	0.778–6.959	0.131
Diabetes, *n* (%)	51 (32.9)	0.874	0.336–2.274	0.782
Dyslipidemia, *n* (%)	98 (63.2)	0.711	0.295–1.715	0.448
Atrial fibrillation, *n* (%)	27 (17.4)	1.185	0.396–3.545	0.761

Lab results				
Uric acid, umol/L, mean (SD)	376.1 (115.0)	1.000	0.997–1.005	0.654
HDL, mmol/L, median (IQR)	1.0 (0.8–1.1)	3.333	0.833–13.334	0.089
TC, mmol/L, median (IQR)	4.7 (3.9–5.3)	0.933	0.656–1.327	0.699
TG, mmol/L, median (IQR)	1.7 (1.1–2.2)	0.937	0.560–1.568	0.804
LDL, mmol/L, median (IQR)	2.9 (2.1–3.3)	0.749	0.484–1.160	0.194
Glycated hemoglobin, %, median (IQR)	6.0 (5.7–6.5)	1.145	0.894–1.466	0.285
D-dimer, mg/L, median (IQR)	0.5 (0.3–1.4)	1.021	0.891–1.169	0.767
Fibrinogen, g/L, median (IQR)	3.6 (3.0–4.2)	1.008	0.854–1.189	0.926
FT3, Median (IQR), pmol/ml	4.3 (4.0–4.5)	0.497	0.172–1.433	0.196
TSH, Median (IQR), nmol/ml	1.8 (0.9–2.1)	0.751	0.465–1.211	0.240
FT4, Median (IQR), pmol/ml	15.2 (14.5–16.1)	0.907	0.716–1.149	0.419

Treatment procedures				
Anticoagulant, *n* (%)	44 (28.4)	2.064	0.855–4.981	0.107
Antiplatelet, *n* (%)	139 (89.7)			0.228^*∗*^
Statin use, *n* (%)	148 (95.5)			0.596^*∗*^
Antihypertensive drugs, *n* (%)	87 (56.1)	1.452	0.579–3.638	0.427
Huoxiang Zhengqi Pill treatment, *n* (%)	77 (49.7)	0.253	0.085–0.757	0.014

^*∗*^Fisher's exact test

## Data Availability

The study data that underlie the results of this article will be given to investigators within two months of the committee's approval of the research proposal.
